# Surfactant-Tuned Phase Structure and Morphologies of Cu_2_ZnSnS_4_ Hierarchical Microstructures and Their Visible-Light Photocatalytic Activities

**DOI:** 10.1186/s11671-017-1868-4

**Published:** 2017-03-09

**Authors:** Yaxin Guo, Jie Wei, Yalong Liu, Tiantian Yang, Zhuo Xu

**Affiliations:** Electronic Materials Research Laboratory, Key Laboratory of Ministry of Education & International Center for Dielectric Research, Xi’an Jiaotong University, 710049 Xi’an, People’s Republic of China

**Keywords:** Cu_2_ZnSnS_4_, Hierarchical microstructure, Kesterite, Wurtzite, Photocatalysis

## Abstract

Cu_2_ZnSnS_4_ (CZTS) hierarchical microstructures were synthesized by using a facile and nontoxic hydrothermal route, which were characterized by X-ray powder diffraction (XRD), scanning electron microscope (SEM), Raman spectra, and UV–Vis absorption spectra. The results and analysis show that surfactants used in the hydrothermal process have significant effect on the phase structures, morphologies, and photocatalytic activities of CZTS powders. Especially, the well-crystallized and pure kesterite CZTS hierarchical microstructures were synthesized with the addition of high-concentration tartaric acid (TA) in the hydrothermal process. A nucleation–dissolution–recrystallization mechanism was discussed, and the photocatalytic activities of CZTS hierarchical microstructures for the degradation of rhodamine B (RhB) were also evaluated. We argue that the crystalline structure and particle morphology have played key roles on the photocatalytic properties of CZTS crystals. A considerably high photocatalytic efficiency of 51.66% after 4 h irradiation was obtained in pure kesterite CZTS hierarchical microstructures, which suggests that CZTS would be a promising candidate of photocatalyst.

## Background

In recent years, it is possible to take the resources and environmental factors into accounts due to the emergence of solar cells [1–3]. Except for silicon-based solar cells, the CuIn_*x*_Ga_1 − *x*_S(Se)_2_ (CIGS) thin-film solar cell has attracted much attention since its fine stability and potential power conversion efficiency. However, the employment of expensive materials such as indium and/or gallium increases the production costs and limits the mass production of these materials. More recently, Cu_2_ZnSnS_4_ (CZTS) was intensely studied and expected to be the most promising candidate to replace Cu(In,Ga)Se_2_ (CIGS) [4, 5], in which raw materials used are low cost, less toxic, and earth abundant. Moreover, CZTS also has a direct band gap of 1.4–1.5 eV with high absorption coefficient in the visible range (>10^4^ cm^−1^) [6–8].

Although a series of traditional physical techniques have been employed for preparing CZTS thin films or solar cells by using pulsed laser deposition, thermal evaporation, and sputtering techniques [9–12], these vacuum-based methods have some innate disadvantages such as complicated equipment, high production costs, and low throughout [13, 14]. To solve these difficulties, many groups have attempted to employ chemical solution routes integrated with nanocrystalline dispersion to fabricate semiconductor films for solar cells at low cost by using low-toxic solvents. Therefore, synthesis of CZTS micro- or nanocrystallines has attracted much attention, and then, many solution-based processes have been proposed, such as hot-injection method, hydrothermal method, solvothermal method, and microwave-irradiated method [15, 16]. Generally, there are two principal crystallographic phase structures of CZTS, which are the so-called stannite (space group $\mathrm{I}\overline{4}2\mathrm{m}$) and kesterite (space group $\mathrm{I}\overline{4}$) [17]. The only difference between the two structures is the arrangement of Cu and Zn atoms. It has been experimentally confirmed that CZTS usually crystallizes in kesterite phase as it is thermodynamically more stable than the stannite one [18]. More recently, a novel wurtzite CZTS nanocrystalline with a hexagonal crystal cell was synthesized by a hot-injection method, which also exhibited the photoelectric response property [19, 20]. Although CZTS nanosheets and nanowires have been studied [21–23], investigation on their photocatalytic activities was scarce [24–26]. More specially, the effect of the crystalline structure and morphology on the photocatalytic activities of CZTS was barely reported.

In this case, by using different surfactants, we reported a low-cost and facile one-pot hydrothermal method to fabricate different phase structures and morphologies of CZTS hierarchical microstructures. The results and analysis show that the crystalline structure, particle size, and morphology of CZTS hierarchical microstructures could be easily tuned by altering the type and concentration of the surfactants. In addition, some key factors on affecting photocatalytic activities of the CZTS hierarchical microstructures were evaluated through the degradation of rhodamine B (RhB).

## Methods

### Syntheses of CZTS Hierarchical Microstructures

Cu(NO_3_)_2_·2H_2_O, Zn(NO_3_)_2_, SnCl_2_·5H_2_O, citric acid (CA), tartaric acid (TA), and ethylene diamine tetraacetic acid (EDTA) were used as raw materials and of analytical grade. Firstly, 1.763 g Cu(NO_3_)_2_·2H_2_O, 1.085 g Zn(NO_3_)_2_, 0.823 g SnCl_2_·5H_2_O, and 1.667 g thiourea, as well as 6.136 g CA, 4,382 g TA, or 10.869 g EDTA, were dispersed in 75-ml deionized water under constant stirring for 2 h, and then, obtained solution was transferred to Teflon-lined stainless steel autoclave of 95-mL capacity. The hydrothermal synthesis was conducted at 200 °C for 48 h in an electric oven. After reaction, the autoclave was naturally cooled down to room temperature. The final product was filtrated and washed with ethanol and distilled water, followed by drying at 70 °C in a vacuum oven for 24 h.

### Photocatalytic Measurement

In this case, the photocatalytic activities of the CZTS hierarchical microstructures were evaluated by the degradation of RhB under visible-light irradiation. A spherical Xe lamp (500 W) with a cutoff filter (*λ* > 420 nm) was used as the visible-light source. In each turn, 20 mg photocatalyst (CZTS powders) was suspended in a 50 mL 0.05 mmol/L RhB aqueous solution, being used for the photocatalytic measurement. Then, this suspension was kept in the dark for 1 h under magnetic stirring to ensure an adsorption–desorption equilibrium between the dye and the catalysts. The relative concentration of RhB in the solution was detected using a UV–Vis spectrophotometer (PerkinElmer Lambda 950 UV/Vis/NIR spectrometer), which is determined by the relative intensity of its absorption peak at 553 nm. The RhB degradation was calculated using the Lambert–Beer equation.

### Characterization

The phase purity and crystalline structure of as-prepared samples was studied with X-ray diffraction (XRD, Philips X’Pert PRO, Cu Kα radiation; *λ* = 1.5405 Ả). The Raman scattering measurement was performed using a Raman microscope (HR800, HORIBA, France) with a laser source of *λ* = 633 nm. The dimension and morphologies of all samples were characterized by field emission scanning electron microscope (FESEM, Quanta F250) with an accelerating voltage of 10 kV.

## Results and Discussion

### Structural Characterization

**Fig. 1:**
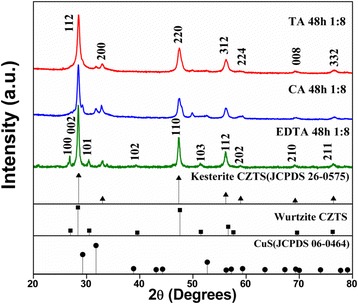
XRD patterns of CZTS hierarchical microstructures synthesized at 200 °C for 48 h with different surfactants, such as ethylene diamine tetraacetic acid (*EDTA*), citric acid (*CA*), and tartaric acid (*TA*). The standard patterns of the kesterite CZTS (JCPDS card no. 26-0575), CuS (JCPDS card no. 06-0464), and the wurtzite one (Ref. [27]) are shown below as reference

In order to investigate the effect of different surfactants and the amount of tartaric acid (TA) on the phase composition of the obtained product, a series of CZTS samples were hydrothermally synthesized by adding different surfactants into the reaction system with a ratio between metal ions and surfactant as 2:1:1:4:8. Figure 1 shows the XRD patterns of as-prepared samples. As seen in Fig. 1, the phase composition of samples strongly depended on the kind of added surfactants. The CZTS sample prepared in the EDTA-dependent reaction system shows a main wurtzite phase, as proposed by Lu et al. [27], although only a small diffraction peak at 33° corresponding to (200) plane of kesterite phase appears. However, the sample obtained from the CA-dependent reaction system suggests two coexisting CZTS phases (wurtzite and kesterite) herein. For example, the diffraction peaks that appeared at about 28°, 33°, 56°, 58°,69°, and 76° are, respectively, attributed to (112), (200), (312), (224), (008), and (332) planes of kesterite CZTS (JCPDS, no. 26-0575). Nevertheless, the doublet peak at about 47° could be decomposed into two individual peaks, which should be corresponding to the (110) plane of wurtzite CZTS and the (220) plane of kesterite one. Of course, a small trace of binary copper sulfide phase is also observed in this sample (such as the tiny peaks at around 29° and 33° should be attributed to CuS phase). More interestingly, the XRD pattern of the sample prepared with TA appears to be a pure kesterite structure.

**Fig. 2:**
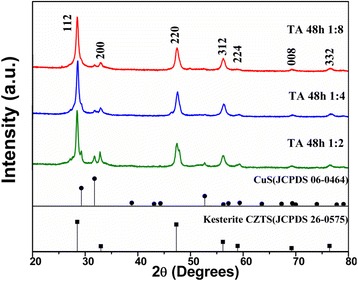
XRD patterns of CZTS hierarchical microstructures synthesized at 200 °C for 48 h with the different amounts of tartaric acid (*TA*), such as 1:2, 1:4, and 1:8. The standard patterns of the kesterite CZTS (JCPDS card no. 26-0575) and CuS (JCPDS card no. 06-0464) are shown below as reference

**Fig. 3:**
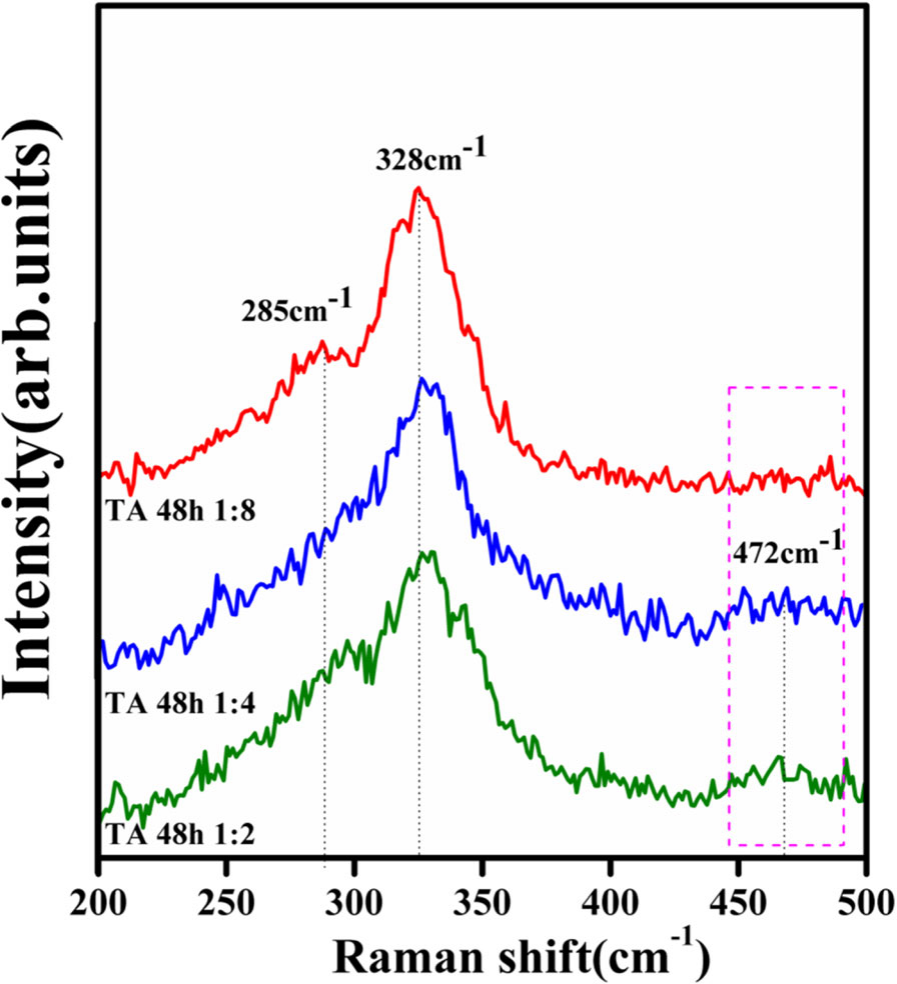
Raman spectra of CZTS hierarchical microstructures obtained at 200 °C for 48 h with the different amounts of tartaric acid (*TA*), such as 1:2, 1:4, and 1:8

The XRD patterns of the CZTS powders obtained with different amounts of TA are shown in Fig. 2. The main diffraction peaks matched well with the kesterite CZTS from the XRD patterns, confirming that the major phase of the sample should be kesterite structure although a little trace of impurity CuS was found. With an increased amount of TA in the reaction system, the main diffraction peaks of kesterite CZTS become narrow and sharp. Meanwhile, the impurity peaks almost disappeared. To further clarify the evolution of phase structure in these samples, Raman scattering was performed. The main peaks observed in Fig. 3 are located at about 285 and 334–338 cm^−1^ corresponding to CZTS, while 472 cm^−1^ corresponding to CuS [28]. Since a weak and broad peak at 472 cm^−1^ was detected, few CuS impurities should be included in the two samples synthesized with low-concentration TA solution (the ratio between CZTS and TA as 1:2 and 1:4). Especially, in the sample synthesized in the high-concentration TA solution (CZTS:TA = 1:8), it is hardly to find any trace of impurities. This is in agreement with the result obtained from the XRD analysis. Therefore, it is rational to speculate that the type and amount of surfactant can adjust the chemical environment, which prevent the phase transition and consequently influence the photocatalytic properties (as shown in the latter).

### Surface Morphology Analysis

**Fig. 4:**
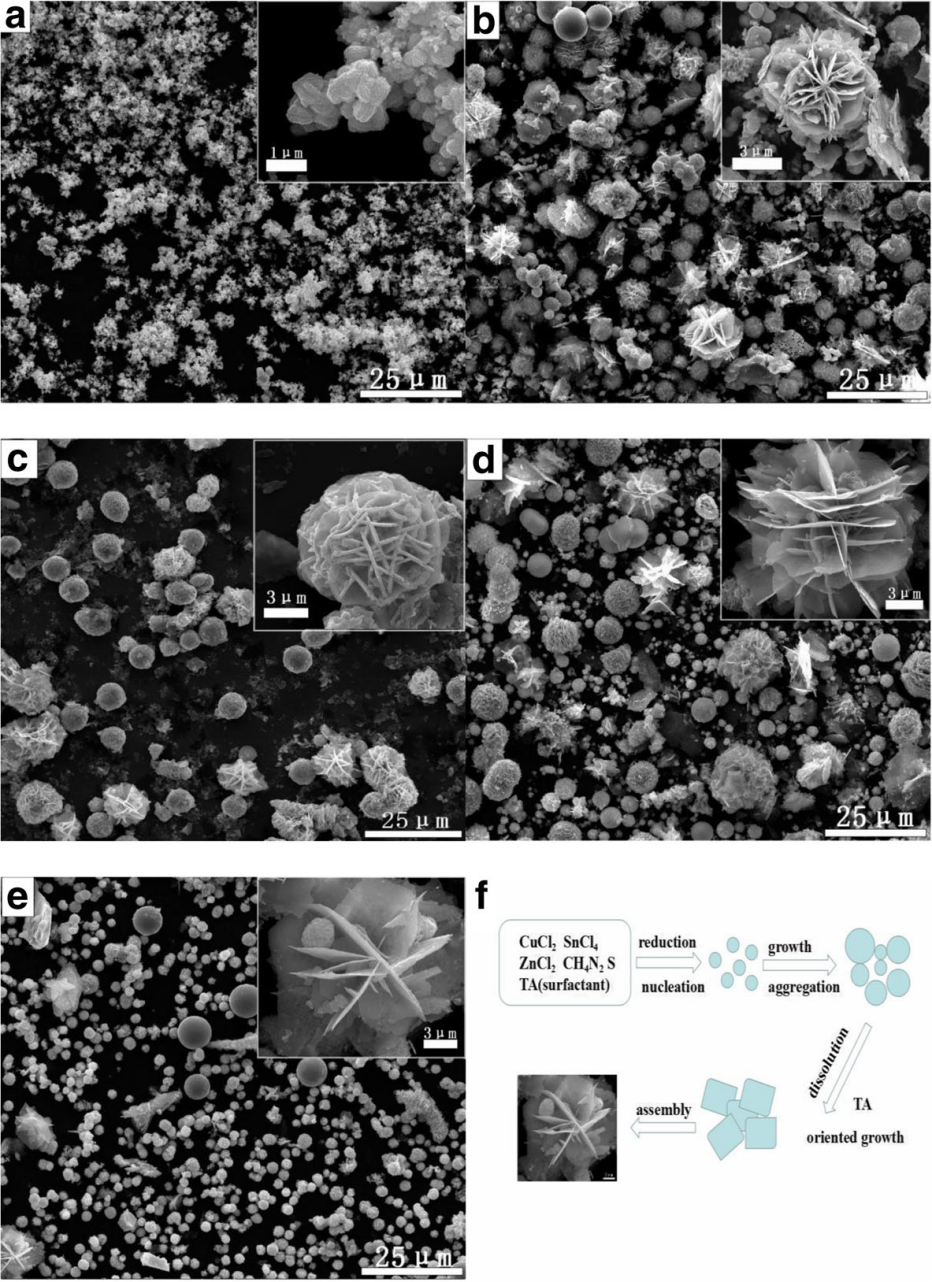
FESEM images of the CZTS particles prepared at 200 °C for 48 h with different surfactants, such as **a** ethylene diamine tetraacetic acid (EDTA), **b** citric acid (CA), and **c** tartaric acid (*TA*), as well as FESEM images of the CZTS particles prepared at 200 °C for 48 h with different amounts of tartaric acid (*TA*), such as 1:2 (**d**), 1:4 (**e**), and 1:8 (**c**). **f** Schematic illustrations of the formation and growth process for flower-like CZTS particles assembled by nanosheets

The morphology of CZTS hierachical microstructures synthesized with different surfactants was investigated by FESEM, as shown in Fig. 4. As synthesized in the EDTA-dependent reaction system, the CZTS are mainly sphere-like particle aggregations with rough surface and about 300–500 nm in diameter (Fig. 4a). From Fig. 4b, it can be observed that CA had intensely influence the morphology and size of the products, which lead to the changes of CZTS particle morphology from sphere-like to flower-like type accompanying with particle size increasing to 5–7 μm. In the sample of CZTS synthesized in the TA-dependent reaction system (Fig. 4c), both sphere-like and flower-like particles with sizes 3–5 μm appeared in the products. These phenomena indicated that the morphology of the as-synthesized CZTS can be easily changed with the participation of different surfactants in the reaction system. Figure 4d, e shows that, by increasing amount of TA, the grain size of CZTS decreased slightly. While the ratio between CZTS and TA is 1:2 (Fig. 4d), the crystal size of CZTS particle aggregations is about 15 μm. As the ratio increased up to 1:4, the quasi-sphere aggregations with a size of 10 μm composed of the nanosheets and sphere-like particle can be obtained, as shown in Fig. 4e. Further increasing the ratio to 1:8 (Fig. 4c), the particle size decreased to about 5 μm. The higher concentration of TA in reaction solution may become a colloid that encapsulates the entire CZTS particle surface and inhibits the growth in all the directions [29]. Inversely, if TA is at a relatively low concentration, it is not possible to completely cover all the crystal planes. TA may preferentially absorb on the face having higher density of surface atoms, thus restricting the growth along those faces [30]. The above results suggest that TA might play two key characters in the reaction: (i) the prevention of particles aggregating and (ii) the selective adhesion to some certain facets of CZTS crystal and controlling the growth rate of these facets [31, 32].

### Growth Mechanism

From the above morphology observation, the formation of flower-like CZTS particles and growth behavior by TA modification might be reasonably expressed as a kinetically controlled nucleation–dissolution–recrystallization process [33], as shown in Fig. 4f. Prior to hydrothermal process, metal ions firstly chelated with thiourea (Tu) in aqueous solution to directly give intermediate (metal-Tu) under continuously stirring.

1 $$ {\mathrm{M}}^{2+}+ n\mathrm{T}\mathrm{u}\leftrightarrow {\left[\mathrm{M}{\left(\mathrm{Tu}\right)}_n\right]}^{2+} $$

At the solvothermal temperature, thiourea (Tu) is attacked by the strong nucleophilic O atoms of H_2_O molecules, which will be broken to produce hydrogen sulfide (H_2_S) slowly [34].

2 $$ {\mathrm{H}}_2{\mathrm{NCSNH}}_2+2{\mathrm{H}}_2\mathrm{O}\to 2{\mathrm{NH}}_3+{\mathrm{CO}}_2+{\mathrm{H}}_2\mathrm{S} $$

In an aqueous medium, H_2_S dissociates as

3 $$ {\mathrm{H}}_2\mathrm{S}\leftrightarrow {\mathrm{H}\mathrm{S}}^{-}+{\mathrm{H}}^{+} $$

4 $$ {\mathrm{H}\mathrm{S}}^{-}\leftrightarrow {\mathrm{S}}^{2-}+{\mathrm{H}}^{+} $$

5 $$ {\left[\mathrm{M}{\left(\mathrm{Tu}\right)}_n\right]}^{2+}+{\mathrm{S}}^{2-}\leftrightarrow \mathrm{CZTS}+ n\mathrm{T}\mathrm{u} $$

During the hydrothermal process, S^2−^ will react with Cu^+^, Zn^2+^, and Sn^2+^ complex to produce CZTS nuclei following with the crystal growth. Based on an Ostwald ripening process, large particles are composed of small ones and they will be incorporated into a solid lattice. At this moment, TA was added and then adhered to the particle surface, while forming the primary nanocrystal. The high surface energy of the inserted particles leads to the thermodynamically instability of the original CZTS microspheres. Simultaneously, partial CZTS nanoparticles are going to disperse and dissolve in the solution and further assemble to platelet-like nanoparticles through oriented aggregation. After a longtime experiment, the CZTS particle crystallization gradually changed from polycrystalline to single crystalline by the grain rotation-induced grain coalescence (GRIGC) mechanism [35]. In the end, hierarchical and flower-like microstructures take the place of the sheet-like nanoparticles.

### Photocatalytic Properties

**Fig. 5:**
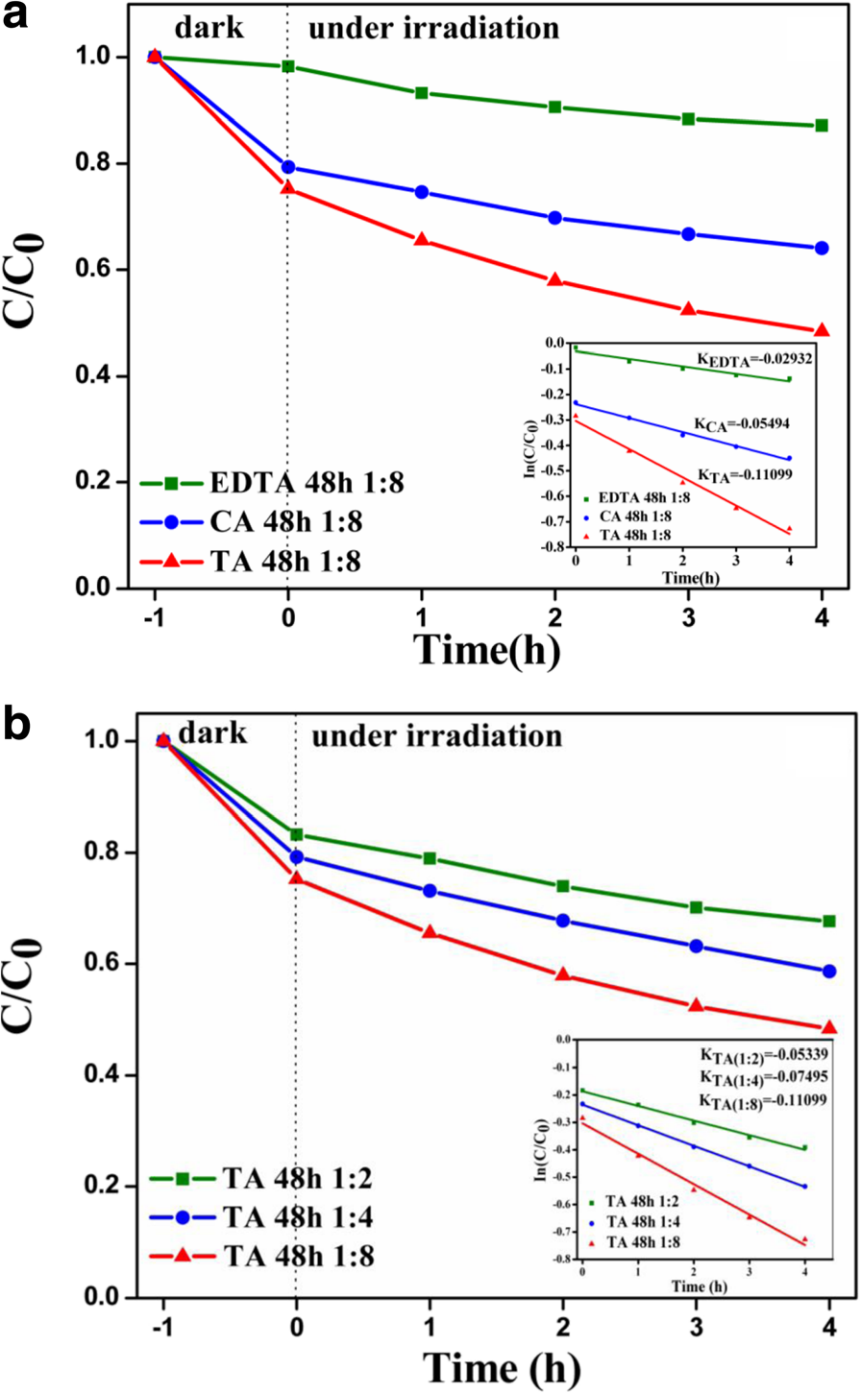
**a** Photocatalytic activities of CZTS samples synthesized with different surfactants and the kinetics of RhB photodegradation over CZTS catalysts (*inset*). **b** Photocatalytic activities of CZTS samples synthesized with different amounts of tartaric acid (*TA*) and the kinetics of RhB photodegradation over CZTS catalysts (*inset*)

The photocatalytic activities of CZTS hierarchical microstructures were evaluated by the photodegradation of 50 mL RhB solution (0.05 mmol/L) under visible-light illumination. Figure 5a presents the photocatalytic activities of CZTS samples synthesized with different surfactants, such as ethylene diamine tetraacetic acid (EDTA), citric acid (CA), and tartaric acid (TA). Before photoreaction, absorption and desorption equilibrium was obtained by mechanical stirring the solution including both RhB and catalysts for 1 h in the dark. In the dark environment, about 2% of RhB was absorbed on the surface of CZTS particles synthesized with EDTA, while 21 and 23% corresponding to the samples synthesized with CA and TA, respectively. The photodegradation of RhB over CZTS samples followed the pseudo-first-order reaction kinetic model: ln(*C/C*_0_) *= kt*, where *k* is the kinetic constant and *C* and *C*_0_ are the current concentration and the initial concentration of RhB, respectively. In a typical degradation process, after visible-light irradiation for 4 h, RhB decomposed only 12.82% with the presence of CZTS samples synthesized with EDTA. However, the degradation efficiencies of RhB rapidly increased to 35.91 and 51.66% with the presence of CZTS samples synthesized with CA and TA, respectively. Inserting Fig. 5a, the graph displays the degradation rate constant (*K*) of RhB over different CZTS photocatalysts. The photodegradation activity sequence for CZTS samples is *K*_TA_(0.11099 h^−1^) > *K*_CA_(0.05494 h^−1^) > *K*_EDTA_(0.02932 h^−1^). The abovementioned XRD and Raman results revealed a structure evolution from wurtzite CZTS synthesized with EDTA to kesterite one synthesized with TA, through an intermediate mixed structure of wurtzite and kesterite CZTS synthesized with CA. Combined with the above photocatalytic analysis, it could be speculated that kesterite CZTS exhibited higher photocatalytic efficiency than wurtzite one. In addition, CZTS hierarchical microstructures synthesized with TA exhibit a higher crystallinity than that with CA [36], which implies fewer defects acting as photo-generated electron–hole recombination centers exist in this kind of CZTS hierarchical microstructures. Therefore, the photocatalytic reaction can occur more efficiently, and then, CZTS hierarchical microstructures synthesized with TA exhibit higher photocatalytic efficiency.

Figure 5b shows the degradation rates of RhB in the presence of CZTS catalysts synthesized with different amounts of TA. The dark experiment demonstrated that the physical absorption rate of RhB was around 16, 20, and 23%, which correspond to three CZTS samples synthesized with the ratio between CZTS and TA as 1:2, 1:4, and 1:8, respectively. Under visible-light irradiation, it can be seen that the photocatalytic efficiency increased with the increasing amount of TA used in the hydrothermal process and the photocatalytic efficiency of RhB over CZTS synthesized at the ratio of 1:8 reached up to 51.66% in 4 h. These results are further confirmed by the degradation rate constant (*K*) of RhB diagram (inset of Fig. 5b), in which the highest degradation rate constant (*K*) up to 0.11099 h^−1^ can be found for the CZTS synthesized with the ratio between CZTS and TA as 1:8. The SEM images revealed a reduced tendency in the particle size of CZTS against the increasing amount of TA used in the synthesis process. The results suggest that the increase of surface area could increase the number of active sites, which will contribute to the promotion of the separation efficiency of the electron–hole pairs in photocatalytic reactions, thus leading to a higher photocatalytic activity. In addition, irregular nanosheet aggregations can allow multiple reflections of the visible light to further enhance the light harvesting pigment, thereby increasing the number of photo-generated electrons and holes that may be involved in the photocatalytic reaction.

**Fig. 6:**
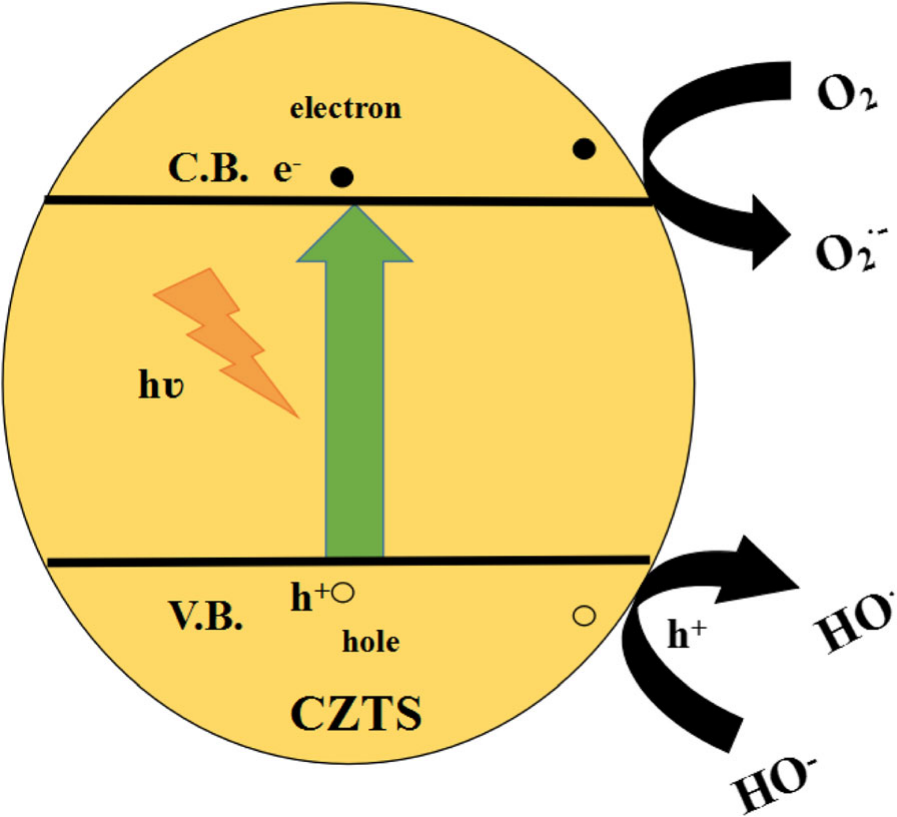
Schematic of CZTS band structure and photocatalytic reaction process under visible-light illumination

Based on the above analysis and discussion, we proposed a possible schematic for the photocatalytic degradation of RhB over CZTS in Fig. 6. Related reaction equations list as follows:

6 $$ \mathrm{CZTS}+\mathrm{h}\upsilon \to \mathrm{CZTS}+{\mathrm{e}}^{-}+{\mathrm{h}}^{+} $$

7 $$ {\mathrm{H}}_2\mathrm{O}+{\mathrm{h}}^{+}\to \cdot p \mathrm{O}\mathrm{H}+{\mathrm{H}}^{+} $$

8 $$ {\mathrm{OH}}^{-}+{\mathrm{h}}^{+}\to \cdot p \mathrm{O}\mathrm{H} $$

9 $$ \mathrm{R}\mathrm{h}\mathrm{B} + \cdotp \mathrm{O}\mathrm{H}\to \mathrm{degradation}\kern0.5em \mathrm{products} $$

10 $$ \mathrm{R}\mathrm{h}\mathrm{B}+{\mathrm{h}}^{+}\to \mathrm{degradation}\kern0.5em \mathrm{products} $$

Since CZTS is a p-type semiconductor, holes are major carries, which are suited for oxidizing organic compounds. Under visible-light irradiation, electrons (e^−^) are excited from the valance band (VB) to the conduction band (CB) of CZTS, causing the generation of holes (h^+^) in the VB simultaneous [Eq. (6)]. Then, the holes (h^+^) may be involved in the process of producing ·OH radicals in the VB by reaction with H_2_O or OH− [Eqs. (7) and (8)]. It is known that hydroxyl radicals (·OH) is considered to be the main reactive species on photocatalytic degradation of organic pollutants in the process. Meanwhile, the holes and hydroxyl radicals can also oxidize RhB into degradation products [Eqs. (9) and (10)].

## Conclusions

In conclusion, a facile surfactant-assisted hydrothermal approach was employed to synthesize CZTS hierarchical microstructures with the different phases (wurtzite and kesterite) and the different morphologies. The analysis of the XRD patterns and Raman spectra indicates that the addition of three surfactants (EDTA, TA, and CA) into reaction system leads to the formation of three CZTS phases (wurtzite, kesterite, or two phases coexisting). Moreover, the well-crystallized and pure kesterite CZTS samples were synthesized when the high-concentration TA was used in the hydrothermal process. The results and analysis show that the concentration of TA intensely influences and controls the phase purity, the size, and the morphology of the final products. A nucleation–dissolution–recrystallization mechanism was proposed for the organization and growth of the flower-like CZTS microstructures assembled by nanosheets. The photocatalytic activities of the different CZTS samples were evaluated by the degradation of RhB under visible-light irradiation. The photocatalytic results indicate that the photocatalytic property of CZTS strongly depends on the phase structure, morphology, and surface area. A considerably high photocatalytic efficiency of 51.66% after 4 h irradiation was obtained in a kind of kesterite CZTS hierarchical microstructures, suggesting that CZTS is indeed a promising photocatalyst being worthy of further study.
